# Immunosuppressive Microenvironment and Efficacy of PD-1 Inhibitors in Relapsed/Refractory Classic Hodgkin Lymphoma: Checkpoint Molecules Landscape and Macrophage Populations

**DOI:** 10.3390/cancers13225676

**Published:** 2021-11-12

**Authors:** Artem Gusak, Liudmila Fedorova, Kirill Lepik, Nikita Volkov, Marina Popova, Ivan Moiseev, Natalia Mikhailova, Vadim Baykov, Alexander Kulagin

**Affiliations:** RM Gorbacheva Research Institute of Pediatric Oncology, Hematology and Transplantation, Pavlov First Saint Petersburg State Medical University, L’va Tolstogo Str. 6-8, 197022 Saint Petersburg, Russia; lepikkv@gmail.com (K.L.); warlord926@gmail.com (N.V.); marina.popova.spb@gmail.com (M.P.); moisiv@mail.ru (I.M.); bmt.lymphoma@gmail.com (N.M.); baikov02@mail.ru (V.B.); kulagingem@rambler.ru (A.K.)

**Keywords:** relapsed or refractory classic Hodgkin lymphoma, nivolumab, immune checkpoints, macrophages

## Abstract

**Simple Summary:**

Classic Hodgkin lymphoma contains rare malignant Hodgkin/Reed–Sternberg cells and abundant reactive populations in the tumor microenvironment. Many aspects of the interaction between tumor cells and immune cells remain unclear. Nevertheless, the microenvironment is believed to play a crucial role in tumor resistance and progression. Current knowledge about the role and dynamics of the tumor microenvironment in Hodgkin lymphoma during anti-PD-1 treatment is limited. The aim of this study was to identify possible predictive and prognostic morphological markers in the treatment of patients with relapsed or refractory classic Hodgkin lymphoma treated with nivolumab and to assess the variability of reactive cell populations after nivolumab therapy. The study was aimed to optimize therapeutic strategy in patients with relapsed or refractory classic Hodgkin lymphoma.

**Abstract:**

To date, the impact of the tumor microenvironment on the prognosis of patients with classic Hodgkin lymphoma (cHL) during anti-PD-1 therapy has been studied insufficiently. This retrospective study included 61 primary samples of lymph nodes from patients who had relapsed/refractory (r/r) cHL and were treated with nivolumab. Repeated samples were obtained in 15 patients at relapse or disease progression after immunotherapy. Median follow-up was 55 (13–63) months. The best overall response rate and progression-free survival (PFS) were analyzed depending on the expression of CD68, CD163, PD-1, LAG-3, TIM-3, CTLA-4, TIGIT, CD163/c-maf in the tumor microenvironment in primary and sequential biopsies. The combination of CD163/c-maf antibodies was used for the identification of M2 macrophages (M2). A low number of macrophages in primary samples was associated with inferior PFS during nivolumab treatment (for CD163-positive cells *p* = 0.0086; for CD68-positive cells *p* = 0.037), while a low number of M2 with higher PFS (*p* = 0.014). Complete response was associated with a lower level of M2 (*p* = 0.011). In sequential samples (before and after nivolumab therapy) an increase in PD-1 (*p* = 0.011) and LAG-3 (*p* = 0.0045) and a depletion of CD68 (*p* = 0.057) and CD163 (*p* = 0.0049)-positive cells were observed. The study expands understanding of the cHL microenvironment structure and dynamics during nivolumab therapy in patients with r/r cHL.

## 1. Introduction

Classic Hodgkin lymphoma (cHL) is a B-cell-derived lymphoid tumor characterized by remarkable clinical and biological features. From the clinical point of view, cHL is a potentially curable disease with 70–80% of patients achieving a long-term remission after a first-line therapy [1,2]. Up to 50% of patients with relapsed and refractory (r/r) disease can be cured or have a prolonged tumor response after autologous hematopoietic stem cell transplantation (ASCT) [3]. However, the prognosis of patients with r/r cHL in case of ASCT failure was unfavorable [4] until new agents, brentuximab vedotin and PD-1 inhibitors, became available [5,6,7]. Nivolumab and pembrolizumab were demonstrated efficacy in patients with r/r cHL in clinical trials as well as in real clinical practice, and significantly improved the outcomes of patients [6,7,8].

Classic HL is a unique histological entity characterized by rare tumor cells and a predominant and diverse tumor microenvironment (TME). The reactive populations surrounding the tumor cells are a key biological factor of cHL carcinogenesis and progression. Hodgkin and Reed-Sternberg (HRS) cells interact with TME cells such as lymphocytes, macrophages, eosinophils, neutrophils, plasma cells, fibroblasts, mast cells, and dendritic cells modulating TME to inhibit an effective immune response [9,10].

Among immune cells recruited to the microenvironment by HRS cells, tumor-infiltrating T cells are one of the most common cell populations. The T cell composition consists of CD8-positive cytotoxic T lymphocytes (CTLs), CD4-positive T-helper cells (THs), and CD4-positive regulatory T cells (Tregs) [11]. The majority of T cells in cHL acquire immunosuppressive functions and their differentiation is shifted towards either THs or Tregs by tolerogenic cytokines (in particular, galectin-1, macrophage migration inhibitory factor, IL-7, CCL17/TARC, CCL22, CCL5, IL-4, IL-5, IL-10, and IL-13) secreted by tumor and non-tumor cells. Moreover, the effector functions (cytotoxicity, cytokine production) of CTLs and their ability to proliferate are reduced, and this phenomenon, known as exhaustion, is associated with high expression of co-inhibitory molecules (PD-1, LAG-3, TIM-3, TIGIT, CTLA-4) on T-cells [12,13,14,15,16].

Tumor-associated macrophages (TAMs) are another abundant cell population educated by HRS cells to become immunosuppressive tumor assistants [11]. An extent of macrophage infiltration can contribute to tumor progression or resistance to therapy. To date, most studies analyzing the role of TAMs in cHL have relied on the identification of macrophages by gene expression profile analysis and/or expression of surface markers (CD68, CD163) with immunohistochemical staining. Multiple studies have shown that an increased number of TAMs was strongly associated with a poor outcome and inferior survival in patients with cHL treated with chemotherapy [17,18,19]. It is important to note that macrophages are a heterogeneous cell population with variable functional activity depending on polarization status. Macrophages are commonly divided into two subsets: classic M1 cells (M1), induced by TH1 cytokines, such as IFN-γ and TNF-α, and alternative M2 cells (M2), polarized by TH2 cytokines IL-4, IL-13, and IL-10. M1 macrophages have anti-tumoral activity and secrete proinflammatory cytokines while M2 deprived of cytotoxic activity may have pro-tumoral effects and produce anti-inflammatory cytokines. M2 facilitate tumor survival, proliferation, progression, and angiogenesis [20,21]. In cHL, HRS cells and T cell composition (TH2, Tregs, exhausted CTLs) help to polarize macrophages toward M2 through the production of cytokines, such as IL-4, IL-10, IL-13, CCL20, CCL22, and lactic acid [11,22]. An increased number of M2 in the TME is associated with inferior survival in patients with cHL treated with chemotherapy [23]. M2 cells may play a critical role in tumor immune escape and immunosuppression [22,23,24,25].

One of the challenges for the introduction of predictive/prognostic factors into clinical practice is that their significance varies according to the type of applied therapy. Numerous publications dwelt upon the impact of tumor cells and TME features as predictive and prognostic markers of chemotherapy efficacy [26,27,28,29,30,31]. Currently, studies on the morphology/cell composition of cHL during immunotherapy are limited, although research data in this area is expanding [32,33,34]. At the same time, the mechanisms of anti-PD-1/PD-L1 therapy in cHL are still not fully elucidated. The relationship between the PD-L1/PD-1 axis and macrophage polarization suggests that PD-1-positive TAMs may be a potential target and directly involved in the antitumor response during immunotherapy [24,35].

This manuscript describes the expression of CD68, CD163, LAG-3, TIGIT, CTLA-4, TIM-3, PD-1, and CD163/c-maf in patients with r/r cHL before and after treatment with anti-PD-1 therapy (nivolumab). We have examined M2 macrophage polarization using double-labeling immunohistochemistry and the expression of co-inhibitory molecules on T-cells in the TME. The aim was to investigate the clinical outcome of patients with r/r cHL who were treated with PD-1 inhibitors depending on the proportion of positive cells for CD68, CD163, PD-1, LAG-3, TIM-3, CTLA-4, TIGIT, and the number of M2 in TME. We also analyzed a variation of these parameters between primary and repeated biopsies after nivolumab failure.

## 2. Materials and Methods

### 2.1. Patients and Tissue Specimens

This study included retrospective data of patients with r/r cHL treated with nivolumab 3 mg/kg or 40 mg flat dose from February 2016 to March 2021 at RM Gorbacheva Research Institute, Pavlov University. The dose of nivolumab varied due to the ongoing clinical study of the nivolumab 40 mg efficacy [36]. At the time of analysis, the median follow-up was 55 (13–63) months.

The analysis included primary (before nivolumab treatment) tumor specimens of 61 patients with r/r cHL taken at initial diagnosis. In 15 patients treated with nivolumab, control lymph node biopsies were performed at disease relapse or progression (repeated samples) after initial nivolumab treatment. The diagnosis was reviewed and confirmed by two expert hematopathologists using morphological and immunohistochemistry methods according to the World Health Organization classification [37]. Routine immunohistochemical panel included staining for CD30 (clone JCM182, BOND RTU, Leica Biosystems Newcastle Ltd, Newcastle Upon Tyne, UK), Pax-5 (clone 1EW, BOND RTU, Leica Biosystems Newcastle Ltd, Newcastle Upon Tyne, UK), CD45 (clone X16/99, BOND RTU, Leica Biosystems Newcastle Ltd, Newcastle Upon Tyne, UK), CD20 (clone L26, BOND RTU, Leica Biosystems Newcastle Ltd, Newcastle Upon Tyne, UK), CD15 (clone MMA, 1:100, Leica Biosystems Newcastle Ltd, Newcastle Upon Tyne, UK). Moreover, staining for PD-L1 (clone 73–10, BOND RTU, Leica Biosystems Newcastle Ltd, Newcastle Upon Tyne, UK), Oct-2 (clone OCT-207, BOND RTU, Leica Biosystems Newcastle Ltd, Newcastle Upon Tyne, UK), BOB-1 (clone TG14, BOND RTU, Leica Biosystems Newcastle Ltd, Newcastle Upon Tyne, UK), Granzyme B (clone 11F1, BOND RTU, Leica Biosystems Newcastle Ltd, Newcastle Upon Tyne, UK), MUM-1 (clone EAU32, BOND RTU, Leica Biosystems Newcastle Ltd, Newcastle Upon Tyne, UK), Bcl-6 (clone LN22, BOND RTU, Leica Biosystems Newcastle Ltd, Newcastle Upon Tyne, UK) and in situ hybridization for Epstein-Barr virus (EBV) encoded mRNA using EBER Probe (Leica Biosystems Newcastle Ltd, Newcastle Upon Tyne, UK) were used in selected cases for differential diagnosis.

The disease status before nivolumab initiation was assessed by PET-CT using Lugano criteria [38]. The response to nivolumab therapy was assessed every 3 months by PET-CT with Lymphoma Response to Immunomodulatory Therapy Criteria (LYRIC) [39]. Primary chemoresistance was defined as the absence of complete remission after the first-line chemotherapy or relapse within 90 days after the end of initial treatment. Early relapse was defined as a relapse that occurred within 12 months after the end of the initial treatment.

Patients’ characteristics are demonstrated in Table 1.

### 2.2. Immunohistochemistry

Immunohistochemical staining for CD68 (clone PG-M1, 1:100, Agilent Technologies Singapore (International) Pte Ltd, Singapore), CD163 (clone 10D6, 1:400, Leica Biosystems Newcastle Ltd, Newcastle Upon Tyne, UK), PD-1 (clone NAT105, ab52587, 1:50, Abcam plc, Cambridge, UK), LAG-3 (clone EPR20261, ab209236, 1:500, Abcam plc, Cambridge, UK), TIM-3 (clone EPR22241, ab241332, 1:800), CTLA-4 (clone CAL49, ab237712, 1:600, Abcam plc, Cambridge, UK), TIGIT (clone BLR047F, ab243903, 1:200, Abcam plc, Cambridge, UK), c-maf (clone EPR16484, ab199424, 1:125, Abcam plc, Cambridge, UK) expression was performed with an automated staining system (Bond III; Leica Biosystems). Internal and external positive controls were used, in the absence of positive control immunohistological slides were excluded from the study.

Immunostaining was performed on 4 mm thick formalin-fixed, paraffin-embedded tissue sections which were deparaffinized and rehydrated. After antigen retrieval slides were processed with hydrogen peroxidase blocking solution to quench endogenous peroxidase activity. The primary monoclonal antibody was applied at a final concentration in antibody diluent (ab64211, Abcam plc, Cambridge, UK). Slides were incubated with primary antibody, subsequently followed by BOND polymer refine detection for single staining (Leica Biosystems Newcastle Ltd, Newcastle Upon Tyne, UK) or ChromoPlexTM 1 Dual Detection for BOND for double staining (Leica Biosystems Newcastle Ltd, Newcastle Upon Tyne, UK). Optimal IHC protocol parameters are shown in Table S1.

The number of total macrophages in all patients was assessed by CD163 expression. A scavenger receptor CD163, expressed almost exclusively on circulating monocytes and tissue macrophages, is considered a more specific marker of TAMs compared to CD68 [40]. However, CD163 or CD68 are not specific markers to determine the polarization status of TAMs. To identify M1- or M2-polarized macrophages, the combined determination of macrophage surface markers (CD68, CD163) with transcriptional factors (STAT1 for M1 or c-maf for M2) can be used [41]. The combination of CD163/c-maf antibodies was used for the identification of M2.

### 2.3. Quantitative Evaluation of Cell Populations

The slides were scanned with Aperio AT2 (Leica Biosystems Imaging, Inc., Vista, CA, USA) and were analyzed by a hematopathologist with ImageScope Analysis software (Leica Biosystems Imaging, Inc., CA, USA) and Qupath (v.0.2.0-m8, open software platform, originally created at the Centre for Cancer Research & Cell Biology at Queen’s University Belfast, developed at the University of Edinburgh, UK, https://qupath.github.io, accessed on 1 April 2021).

For CD68, CD163, PD-1, LAG-3, TIM-3, CTLA-4, and TIGIT, first, we counted manually the proportion of single-positive cells among total cells of TME in a representative area of 0.065 mm^2^ for each scanned slide using the ImageScope Analysis program. At the second step, the same areas were processed in the Qupath program to select parameters allowing for the same positive cell yield with automated analysis. Finally, the whole slide was subjected to automated positive cell count using parameters selected at the second step. Foci of necrosis and fields of fibrosis were excluded from the analyzed area. For M2 (CD163+/c-maf+), the proportion of double-positive cells among total CD163+ cells was calculated manually in the ImageScope Analysis program. The analyzed area was approximately 1.3 mm^2^ (20 fields of view).

### 2.4. Statistical Analysis

The clinical endpoints assessed in this study were best overall response (BOR), overall response rate (ORR), progression-free survival (PFS), overall survival (OS). Overall response rate (ORR) was defined as the proportion of patients with complete response (CR) or partial response (PR) in measurable lesions by LYRIC criteria within a timeframe of 12 months. Progression-free survival was defined as the time from the first nivolumab cycle initiation to disease progression, relapse, or death; OS was defined as the time from the first nivolumab cycle initiation to death due to any reason. In each survival outcome, data were censored at the date of the last contact for patients who have not experienced the events of interest during their follow-up. Progression-free survival was also censored at the date of additional therapy initiation.

The survival was estimated by the Kaplan-Meier method and comparisons were made by log-rank test. The impact of the expression level of IHC markers (CD68, CD163, PD-1, TIM-3, CTLA-4, TIGIT, LAG-3) and the number of M2 on PFS was tested with the Kaplan-Meier method. To investigate differences between groups, non-parametric tests were applied, including the Mann-Whitney signed-rank test, Kruskal-Wallis test, and Spearman rank correlation for independent variables, and Wilcoxon two-sample paired test (*p* ≤ 0.05) for analysis of dependent variables. To define a threshold level of interval variables, a receiver-operating characteristic (ROC) analysis was used. Data analysis was performed using R version 4.0.2 software (R Core Team (2020). R: A language and environment for statistical computing. R Foundation for Statistical Computing, Vienna, Austria. https://www.R-project.org/ accessed on 22 June 2020).

## 3. Results

### 3.1. Clinical Characteristics

**Figure 1:**
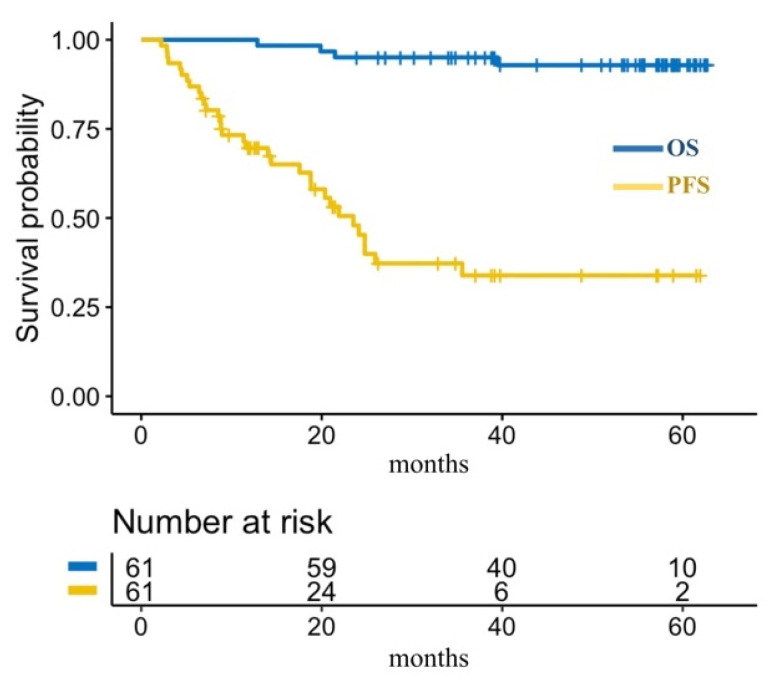
Overall survival (OS) and progression-free survival (PFS).

All 61 patients were included in the treatment efficacy analysis. The overall response rate was 69%. According to LYRIC criteria CR, PR, stable disease (SD), indeterminate response (IR), and progressive disease (PD) as the best response was achieved by 41%, 28%, 2%, 23%, and 7% of patients, respectively. At the data cut-off, the median OS was not reached, 4-year OS was 92.9% (95% CI: 81.9–97.3%) (Figure 1). Median PFS was 23.5 months (95% CI: 17.5–35.6 months) with 4-year PFS of 33.9% (95% CI: 20–48.3%) (Figure 1).

### 3.2. TME Composition

**Figure 2:**
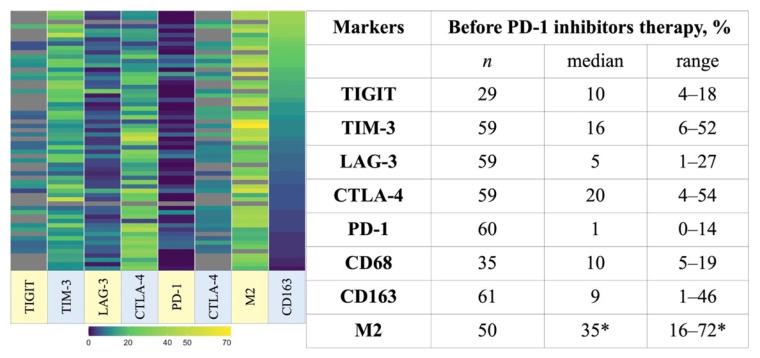
Expression of the assessed markers in the primary biopsies from patients with r/r cHL. * the proportion of single-positive cells among total cells of TME; Abbreviations: M2—M2-macrophages.

The expression level of the assessed markers in each patient’s biopsy was heterogeneous and shown in Figure 2. PD-1, CTLA-4, TIGIT, LAG-3, TIM-3 expression was detected on lymphoid cells in TME while TIM-3 was expressed on a moderate fraction of HRS cells and on multiple macrophages identified by CD68 and CD163. We did not find PD-1 expression on macrophages.

CTLA-4-positive cells were the most abundant cell population (median 20%, range 4–54%) and usually densely surrounded HRS cells. PD-1-positive cells were the smallest cell population (median 1%, range 0–14%), in most samples, the expression was weak or moderate compared to bright expression by follicular THs in residual germinal centers. Expression of PD-1 on immune cells (>1% positive cells) was present in 42% of cases. Expression of LAG-3 on immune cells (>1% positive cells) was observed in 93% of cases. Expression of TIM-3, CTLA-4, TIGIT on immune cells (>1% positive cells) was observed in 100% of cases. PD-L1 expression was analyzed in 19 patients, including those with inferior and superior PFS. In all studied cases, all tumor cells and most macrophages were positive. PD-L1 expression intensity on tumor cells was independent of clinical outcomes (Figure S1).

**Figure 3:**
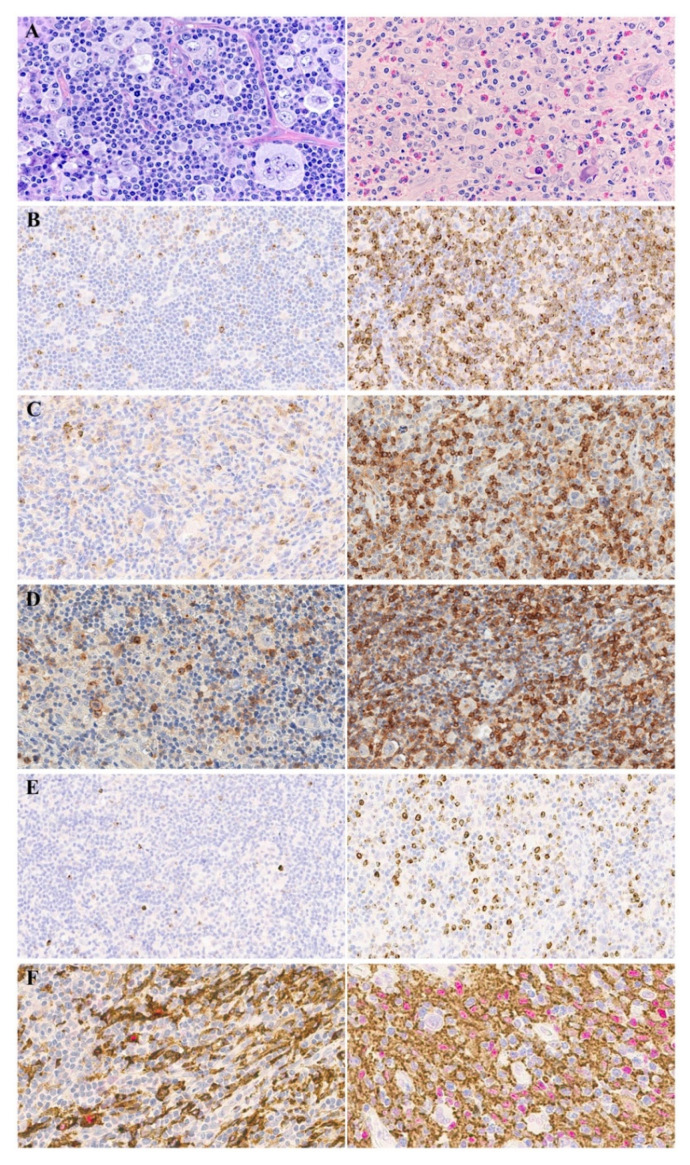
Primary biopsies of patients with r/r cHL. (**A**): The diversity of TME, hematoxylin and eosin staining; (**B**): CTLA-4 expression: left—CTLA-4 low, right—CTLA-4 high; (**C**): TIM-3 expression: left—TIM-3 low, right—TIM-3 high; (**D**): TIGIT expression: left—TIGIT low, right—TIGIT high; (**E**): LAG-3 expression: left—LAG-3 low, right—LAG-3 high; (**F**): CD163/c-maf expression: left—CD163/c-maf low, right—CD163/c-maf high. [Original magnification: (**A**) ×400; (**B**–**F**) ×200].

The diversity of TME and marker expression is demonstrated in Figure 3.

A significant positive correlation was found between the expression level of CD68 and CD163 (correlation coefficient 0.62, *p* < 0.01), CD163 and TIM-3 (correlation coefficient 0.39, *p* < 0.01), PD-1 and LAG-3 (correlation coefficient 0.31, *p* = 0.02). There was a negative correlation between CD163 and CTLA-4 (correlation coefficient −0.49, *p* < 0.01). Details of the correlational analysis are shown in Figure S2.

We examined the relationship between selected clinical features and CD68, CD163, PD-1, TIM-3, CTLA-4, TIGIT, LAG-3 expression, and level of M2. There was no significant association between age, stage, B symptoms, the course of the disease associated with primary resistance or early relapse, ECOG status, histological type of cHL, and studied markers.

### 3.3. The Association of TME Characteristics and Clinical Outcomes

#### 3.3.1. TME and BOR to Nivolumab Therapy

**Figure 4:**
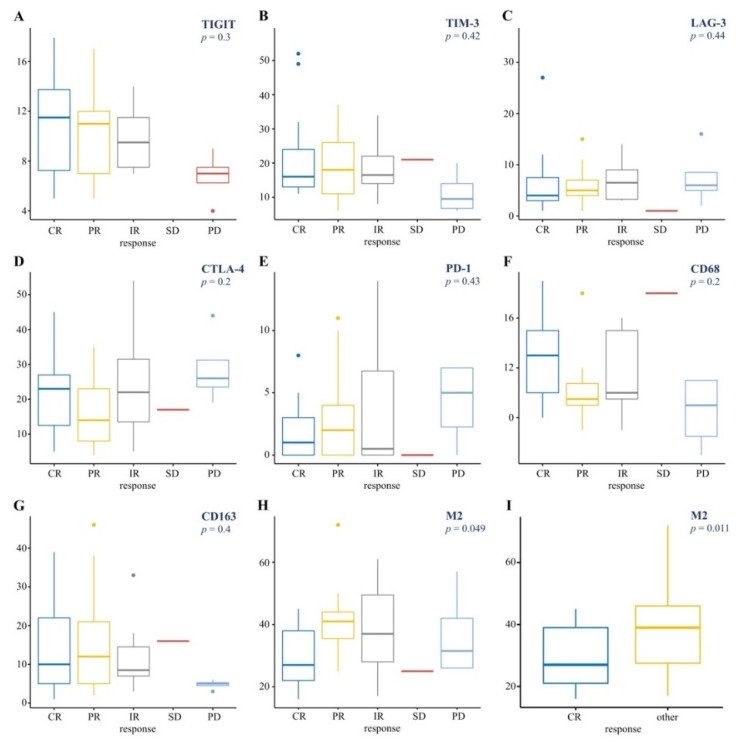
The best overall response to nivolumab depending on the expression of co-inhibitory molecules and proportion of M2-macrophages (M2): (**A**) TIGIT, (**B**) TIM-3, (**C**) LAG-3, (**D**) CTLA-4, (**E**) PD-1, (**F**) CD68, (**G**) CD163, (**H**) M2; (**I**) complete response versus M2. Abbreviations: CR—complete response; PR—partial response; IR—intermediate response; SD—stable disease; PD—progressive disease; other—PR + IR + SD + PD. Boxes indicate first to third quartile; center line, the median; whiskers, range of data points within upper (third quartile + 1.5 × interquartile range) and lower (first quartile − 1.5 × interquartile range) limits; and points, maximum and minimum data point.

There was no statistically significant association between the proportion of PD-1, LAG-3, CTLA-4, TIM-3, TIGIT-positive cells in TME and BOR to nivolumab (*p* > 0.05) (Figure 4A–E). There was also no association between total macrophages number (CD68-positive and CD163-positive cells) and BOR (Figure 4F,G). At the same time, an association between the level of M2 and BOR to nivolumab was demonstrated (*p* = 0.049) (Figure 4H). A low level of M2 was associated with a higher probability of CR (*p* = 0.011) (Figure 4I).

#### 3.3.2. TME and Survival after Nivolumab Therapy

**Figure 5:**
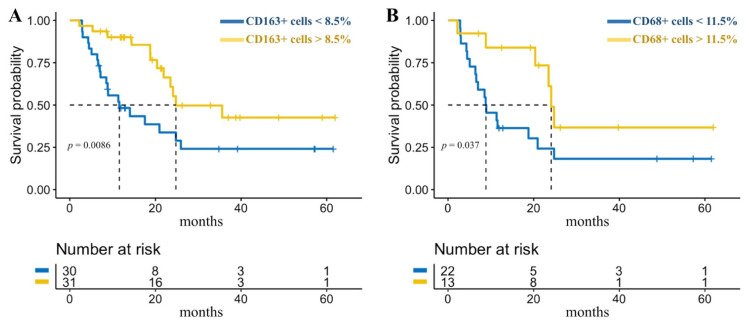
Progression-free survival depending on (**A**) CD163 and (**B**) CD68 expression levels in primary biopsies. The dotted line indicates median of survival.

For descriptive purposes, ROC analysis was applied to define low and high-risk groups with an 8.5% cut-off value of CD163 expression and 11.5% cut-off value of CD68 expression. In the CD163low group, 4-year PFS was 24.12% (95% CI: 9.33–42.62%) with a median PFS of 11.6 months (95% CI: 7.17–24.8 months) while in the CD163high group –42.6% (95% CI: 20.2–63.4%) with a median of 24.8 months (95% CI: 20.37 months–not achieved), *p* = 0.0086. In the CD68low group 4-year PFS was 18.18% (95% CI: 4.9–38.15%) with a median PFS of 8.87 months (95% CI: 5.07–20.9 months) while in the CD68high group –36.71% (95% CI: 9.04–65.85%) with a median of 24.1 months (95% CI: 8.83 month–not achieved), *p* = 0.037 (Figure 5).

The cut-off value for M2 in the ROC analysis was 19.5%. None of the patients with low-level M2 in primary samples progressed after nivolumab treatment at the moment of analysis. In the M2 high-level group 4-year PFS was 24.4% (95% CI: 10.5–41.3%) with a median PFS of 20.9 months (95% CI: 14–24.8 months) and in the M2 low-level group—100% (95% CI: not achieved–not achieved) with a median of PFS was not achieved. Details are shown in Figure S3.

### 3.4. The Dynamics of TME Composition during Immunotherapy

**Figure 6:**
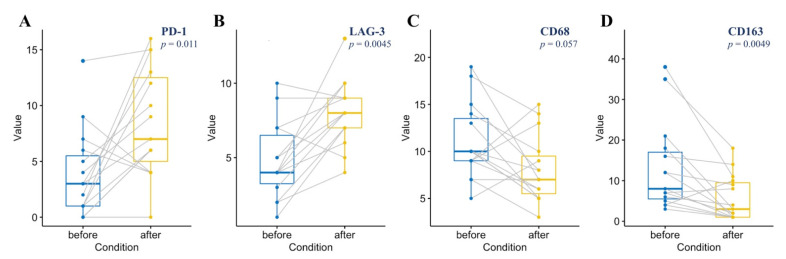
The dynamics of PD-1 (**A**), LAG-3 (**B**), CD68 (**C**), CD163 (**D**) expression (proportion of positive cells) in sequential biopsies of patients with relapsed and refractory classic Hodgkin lymphoma. Boxes indicate first to third quartile; center line, the median; whiskers, range of data points within upper (third quartile + 1.5 × interquartile range) and lower (first quartile − 1.5 × interquartile range) limits; and points, maximum and minimum data point. The connecting lines refer to individual patients’ values.

**Figure 7:**
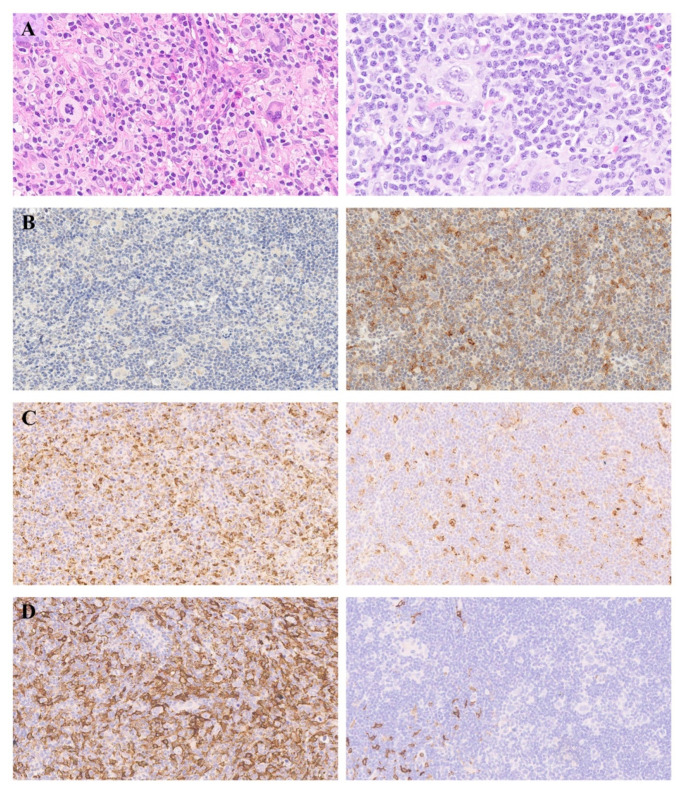
Sequential biopsies of patients with relapsed and refractory classic Hodgkin lymphoma (left—primary biopsy; right—repeated biopsy after progression/relapse during nivolumab treatment. (**A**) dynamics of the tumor microenvironment: decreased level of macrophages, eosinophils, plasma cells in repeated biopsy (representative case), hematoxylin and eosin staining; (**B**) increased level of PD-1-positive cells in repeated biopsy; (**C**) decreased level of CD68-positive cells in repeated biopsy; (**D**) decreased level of CD163-positive cells in repeated biopsy. [Original magnification: (**A**) ×400; (**B**–**D**) ×200].

A statistically significant increase in PD-1 (median 7% vs. 3%, *p* = 0.011) and LAG-3-positive (8% vs. 4%, *p* = 0.0045) T-cells and depletion of CD68 (7% vs. 10%, *p* = 0.057) and CD163-positive (3% vs. 8%, *p* = 0.0049) cells in repeated biopsies after nivolumab treatment was observed compared to the level of expression in primary biopsies (Figure 6A–D and Figure 7A–D. There was no significant difference between primary and repeated biopsies regarding the level of TIM-3, TIGIT, CTLA-4 expression, and the proportion of M2 (*p* > 0.05).

## 4. Discussion

PD-1 inhibitors comprise an effective treatment approach in patients with r/r cHL [6,7]. The concept of anti-PD-1 therapy is based on the almost universal rearrangement of 9p24.1 in cHL, leading to PD-L1 overexpression on tumor cells in nearly all patients [26]. However, about 30% of patients remain refractory or progress during anti-PD-1 therapy, which makes the search for prognostic factors of a durable response highly relevant [8]. The sensitivity of cHL to therapy can be determined both by the features of the tumor cells and/or their microenvironment [26,27,32,33,42]. In addition to the developed PD-L1-PD-1 axis [26], other co-inhibitory molecules, such as TIM-3, TIGIT, CTLA-4, and LAG-3, may play an important role in the development of resistance or failure of antitumor response [15,16,43]. It has been shown that the expression level of these molecules is increased in cHL in comparison with reactive lymphoid tissue [28,29], a state of disease remission [44] or other tumors [45].

For the first time, a broad representative immunohistochemical panel for co-inhibitory molecules was used to describe an immunosuppressive T-cell ecosystem in patients with cHL. We detected that the CTLA-4-positive and TIM-3-positive fractions of the total cells were greater in cHL compared to PD-1, LAG-3, or TIGIT-positive cells, while PD-1-positive cells were a minor population. We found that weak to moderate expression of PD-1 on immune cells (>1% positive cells) was present in 42% of cases. The expression of TIM-3, CTLA-4, LAG-3, TIGIT was moderate or strong and observed in 93–100% cases. This data is comparable to the other studies in which PD-1 expression was observed in less than 40% of cases (range 16–40%) [28,30,46]. TIM-3 expression was observed in 100% of adult cHL cases [47], LAG-3 expression was found in 63% of pediatric cases [48] and in 100% of adult cases [47], TIGIT expression ranged from 56% (cases where cells surrounded HRS) [49] to 100% [50].

Our immunohistochemical analysis demonstrated that HRS cells were frequently in direct contact with macrophages or with cells expressing co-inhibitory molecules. Carey et al. and Patel et al. presented topographic maps of the microenvironmental niche for HRS cells using multiplex immunofluorescence microscopy and confirmed the close relationship between cells expressing co-inhibitory molecules, tumor cells, macrophages, and other potential immunosuppressive players of cHL in TME [51,52]. For example, T cells contacting HRS cells are more frequently positive for CTLA-4 than for PD-1 or LAG-3 [52]. Thus, close relationships between HRS cells, macrophages, and cells expressing checkpoint molecules may create an immunosuppressive niche in TME.

We observed that the number of PD-1-positive cells correlated with the number of LAG-3-positive cells, however, the presence of co-expression of these markers by T cells has not been established. Aoki et al. demonstrated that most PD-1-positive T cells do not co-express LAG-3 [29]. Some authors have found co-expression of LAG-3 and CTLA-4 [29], as well as PD-1 and TIGIT [50] in most T cells. However, a more detailed analysis of the relationship between T cells showed that co-expression between PD-1, LAG-3, CTLA-4-positive cells is heterogeneous. Multiple (>40%) T cell populations were negative for these markers while triple-positive cells comprised only 1% of the total number of T cells. Co-expression of LAG-3 and PD-1 on T cells was observed in 4% of the total number of cells while double-positive cells for other pairs of receptors (CTLA-4 and LAG-3; PD-1 and CTLA-4) were observed in 5% [52].

The prognostic significance of PD-1, CTLA-4, TIM-3, TIGIT, LAG-3 in adult cHL during anti-PD-1 is unclear. In our study, there was no significant association of expression of mentioned markers with the clinical outcome. In previous studies, the impact of PD-1, TIGIT, and LAG-3 expression on clinical outcomes after chemotherapy treatment was analyzed. Several studies and reviews reported that increased numbers of PD-1-positive cells were associated with worse outcomes and shorter survival in cHL patients [28,30,53,54,55,56] while other researchers showed that PD-1 expression had no significant association with survival [57,58]. TIGIT status did not correlate with response to treatment [49]. LAG-3 expression was not associated with survival outcomes in pediatric cases [48] but had prognostic significance in adult patients [29].

Reports about TAMs as a prognostic marker are contradictory. Most authors demonstrated that a high number of TAMs predicted shorter survival after chemotherapy [18], while data regarding the impact of macrophages on the outcome of patients treated with immunotherapy is limited. To identify TAMs, CD163 was chosen as the basic surface marker. In primary biopsies of 61 patients treated with nivolumab, we found that macrophages were associated with a favorable prognosis. Patients with low levels of TAMs in the primary tissue samples showed inferior survival in the study group compared with inverse relationships in patients treated with standard chemotherapy [31]. This association may be explained by the activated PD-1/PD-L1 axis by TAMs. Some studies demonstrated that TAMs express high levels of PD-1 [35] and PD-L1 [59,60]. Moreover, the proportion of pro-tumor M2 among PD-1-positive TAMs was higher than in M1 [35]. However, the immunohistochemistry for detection of the PD-1 expression on TAMs was not a sufficiently sensitive method in comparison with gene expression profile analysis [35]. We did not find PD-1 expression on TAMs, while most macrophages expressed PD-L1. Some reports confirmed that downregulation of PD-L1 expression was a factor for the transformation of immunosuppressive to immunostimulatory macrophages and demonstrated a role of PD-L1 in the M1/M2 polarization [24]. A worse survival was observed in pediatric patients with higher numbers of M2 who were treated with chemotherapy [22]. Using a double-labeled immunohistochemical study we demonstrated that low M2 (CD163+/c-maf+) level was associated with a complete response and better survival. These results suggest that antitumor response mediated by the PD-1/PD-L1 blockade may be partially related to the effect of TAMs.

We examined the immune checkpoints expression and the number of TAMs in a cohort of 15 patients with primary and in repeated samples after immunotherapy failure and found the enrichment of PD-1-positive and LAG-3-positive cells and depletion of TAMs (CD163, CD68-positive cells). In previously published studies, several researchers found increasing levels of PD-1-positive T cells in cHL at relapse after standard chemotherapy by the immunohistochemical method [61], while in other reports, changes in PD-1-positive cell counts at relapse were not found by immunohistochemical or gene expression analysis [62,63]. In the analysis of the specimens at relapse in patients receiving standard chemotherapy, Schnitter et al. found no evidence of enrichment of TAMs (CD163-positive cells) [62]. The expression markers or cell composition may change when patients receive immunotherapy. Saase et al. revealed that the number of PD-1-positive T cells tended to be higher at repeated samples after anti-PD-1 compared to the biopsy before anti-PD-1 therapy while the number of PD-L1 macrophages tended to be lower at repeated samples [64]. However, early dynamics of TME and immunological response after starting immunotherapy differ from the immunological effects observed in the peripheral blood [33,65]. In addition, it remains unclear whether the tumor-reactive cellular populations change after previous non-immunotherapeutic agents.

The limitations of this study include a retrospective study design within a single institution (this may lead to selection bias and a limited number of patients), absence of information on the expression of the corresponding ligands in the receptor–ligand system, EBV status of the tumor, as well as insufficient assessment of the polarization profile of macrophages. There was also no validation cohort to define the cut-off prognostic values of M2.

Our observations underline the relevance of further studies on the role of macrophages as predictive/prognostic markers for anti-PD-1 therapy in cHL. This direction of research may facilitate the development of a background for new approaches to the treatment of r/r cHL. The abundant composition of macrophages with potential PD-1 or PD-L1 expression may help to explain the favorable effect of anti-PD-1 therapy. The study expands the knowledge on the composition and significance of the microenvironment in cHL and shows the decisive importance of quantitative and qualitative parameters of macrophages in relation to the effectiveness and development of resistance to immunotherapy.

## 5. Conclusions

In this study a comprehensive immunohistochemical analysis of the microenvironment was performed, including evaluation of the wide spectrum of co-inhibitory receptors expression on immune cells, the number of macrophages, as well as a partial assessment of the polarization profile of macrophages and the impact of these markers on treatment outcomes. Using a cohort of patients with long-term follow-up, we found that a low number of TAMs in primary samples was associated with inferior PFS, while a low level of M2 macrophages was correlated with a complete response and better survival. The dynamics of cell composition in repeated biopsies after anti-PD-1 therapy was demonstrated. In control biopsies after nivolumab treatment, the cell profile in the tumor microenvironment was altered. The number of PD-1-positive T cells and LAG-3-positive T cells increased while the number of macrophages decreased.

## Figures and Tables

**Table 1:** Patient’s characteristics.

Characteristics	*n* = 61
Age, median age (range)	34 (14–53)
**Histological type of cHL, *n* (%)**	
Nodular sclerosis	51 (84)
Mixed cellularity	8 (13)
Lymphocyte-rich	2 (3)
**Disease stage at diagnosis, *n* (%)**	
I	1 (2)
II	27 (44)
III	10 (16)
IV	23 (38)
B-symptoms at diagnosis, *n* (%)	38 (62)
Bulky disease at diagnosis, *n* (%)	7 (11)
Refractory disease, *n* (%)	39 (64)
Early relapse, *n* (%)	8 (13)
Prior ASCT, *n* (%)	17 (28)
Prior brentuximab vedotin, *n* (%)	27 (44)
Prior radiotherapy, *n* (%)	41 (67)
Prior lines of therapy before nivolumab, median (range)	5 (2–10)
Extranodal involvement at nivolumab initiation, *n* (%)	39 (64)
B-symptoms at nivolumab initiation, *n* (%)	33 (54)
**ECOG status at nivolumab initiation, *n* (%)**	
0	8 (13)
1	30 (49)
2	16 (26)
3	7 (12)
**Nivolumab dose, (%)**	
40 mg	13 (21)
3 mg/kg	48 (79)
Number of nivolumab cycles, median (range)	21 (1–48)

Abbreviations: cHL—classic Hodgkin lymphoma, ASCT—autologous stem cell transplantation, ECOG status—Eastern Cooperative Oncology Group (ECOG) performance status.

## Data Availability

The data presented in this study are available on request from the corresponding author.

## Supplementary Materials

The following are available online at https://www.mdpi.com/article/10.3390/cancers13225676/s1, Figure S1: PD-L1 expression in representative cases with different progression-free survival (PFS), primary biopsies: (A) PFS of 61 months, (B) PFS of 2.9 months, Figure S2: Correlation between assessed markers in primary biopsies, Figure S3: Progression-free survival depending on M2 level in primary biopsies, Table S1: Optimal immunohistochemical protocol parameters.
