# Supplementary material for: Immunosuppressive Microenvironment and Efficacy of PD-1 Inhibitors in Relapsed/Refractory Classic Hodgkin Lymphoma: Checkpoint Molecules Landscape and Macrophage Populations
Source: Cancers (Basel). 2021 Nov 12;13(22):5676. doi: 10.3390/cancers13225676 (PMC8616219; doi:10.3390/cancers13225676)
Supplement: Supplementary file 1 [file cancers-13-05676-s001.zip › cancers-1424218-supplementary.pdf]

Supplementary Materials

Immunosuppressive Microenvironment and Efficacy of PD-1 Inhibitors in Relapsed/Refractory Classic Hodgkin Lymphoma: Checkpoint Molecules Landscape and Macrophage Populations

Artem Gusak, Liudmila Fedorova, Kirill Lepik, Nikita Volkov, Marina Popova, Ivan Moiseev, Natalia Mikhailova, Vadim Baykov and Alexander Kulagin

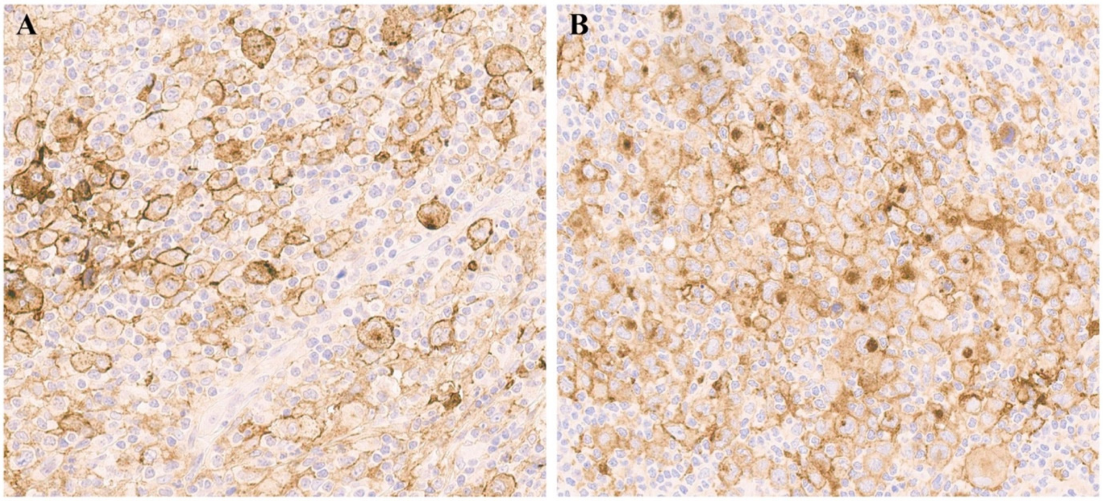

Figure S1. PD-L1 expression in representative cases with different progression-free survival (PFS), primary biopsies: (**A**) PFS of 61 months, (**B**) PFS of 2.9 months. [Original magnification: (**A,B**) ×400].

	PD-1	LAG-3	CTLA-4	TIM-3	TIGIT	CD68	CD163	M2
**M2**								$r = 1$
**CD163**							$r = 1$	$r = 0.2$, $p = 0.17$
**CD68**						$r = 1$	$r = 0.62$, $p < 0.01$	$r = 0.14$, $p = 0.47$
**TIGIT**					$r = 1$	$r = 0.16$, $p = 0.4$	$r = 0.25$, $p = 0.19$	$r = 0.01$, $p = 0.98$
**TIM-3**				$r = 1$	$r = 0.28$, $p = 0.14$	$r = 0.22$, $p = 0.21$	$r = 0.39$, $p < 0.01$	$r = 0.05$, $p = 0.73$
**CTLA-4**			$r = 1$	$r = -0.21$, $p = 0.12$	$r = -0.07$, $p = 0.7$	$r = -0.17$, $p = 0.35$	$r = -0.49$, $p < 0.01$	$r = -0.18$, $p = 0.2$
**LAG-3**		$r = 1$	$r = 0.1$, $p = 0.44$	$r = 0.16$, $p = 0.23$	$r = 0.01$, $p = 0.97$	$r = 0.16$, $p = 0.38$	$r = 0.04$, $p = 0.75$	$r = 0.01$, $p = 0.93$
**PD-1**	$r = 1$	$r = 0.31$, $p = 0.02$	$r = 0.23$, $p = 0.08$	$r = 0.02$, $p = 0.87$	$r = 0.3$, $p = 0.11$	$r = 0.05$, $p = 0.77$	$r = -0.08$, $p = 0.53$	$r = 0.08$, $p = 0.57$

Figure S2. Correlation between assessed markers in primary biopsies. Abbreviations: M2–M2-macrophages, *r*–correlation coefficient.

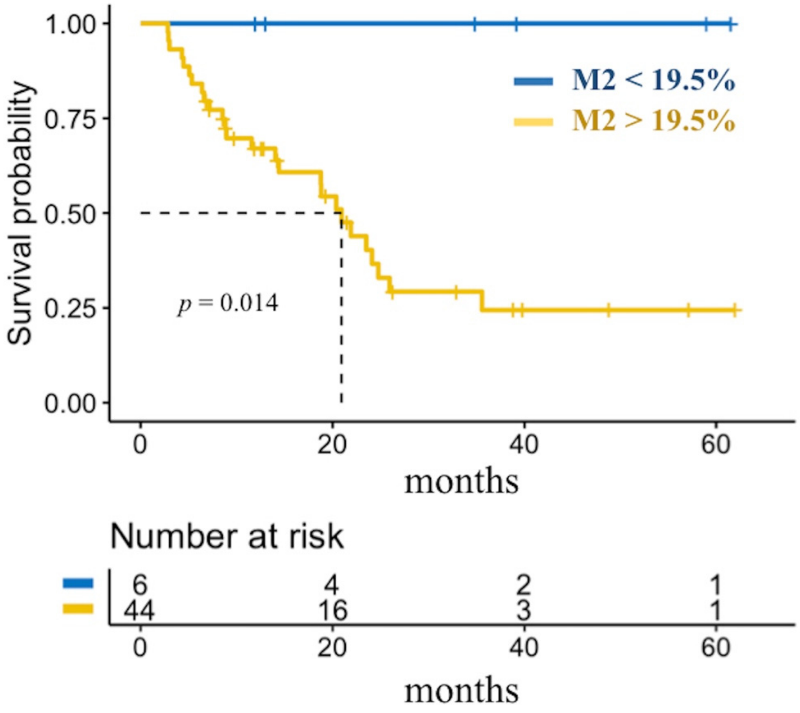

Figure S3. Progression-free survival depending on M2 level in primary biopsies. Abbreviations: M2–M2-macrophages. The dotted line indicates median of survival.

Table S1. Optimal immunohistochemical protocol parameters.

**Immunohistochemistry of Single Staining**									
**Antibody**	**Clone**	**C**	**AR, T, Min, ER1/2**	**PB, Min**	**PrAb, Min**	**PP, Min**	**Pol, Min**	**DAB, Min**	**H, Min**
CD68	PG-M1, Dako	1:100	100°C, 30, ER2	5	15	8	8	10	5
CD163	10D6, Leica	1:400	100°C, 30, ER1	5	15	8	8	10	5
PD-1	NAT105, ab52587	1:50	100°C, 30, ER2	5	20	8	8	10	5
LAG-3	EPR20261, ab209236	1:500	100°C, 30, ER2	5	30	8	8	10	5
TIM-3	EPR22241, ab241332	1:800	100°C, 30, ER2	5	30	8	8	10	5
TIGIT	BLR047F, ab243903	1:200	100°C, 30, ER1	5	30	8	8	10	5
CTLA-4	CAL49, ab237712	1:600	100°C, 30, ER2	5	30	8	8	10	5
**Immunohistochemistry of double staining**									
**Antibody**	**Clone**	**C**	**AR, T, Min, ER1/2**	**PB, Min**	**PrAb, Min**	**mHRP, Min**	**rAP, Min**	**DAB/Red, Min**	**H, Min**
Antibody Cocktail: CD163/c-maf	10D6, Leica EPR16484, ab199424	1:125	100°C, 40, ER2	5	30	8	20	10/ 5	5

Abbreviations: C–concentration, AR–antigen retrieval, PB–peroxidase block, PrAb–primary antibody, PP–post primary, Pol–polymer, mHRP–mouse horseradish peroxidase, rAP–rabbit alkaline phosphatase, DAB–3,3'-diaminobenzidine, H–hematoxylin.
